# The Role of Diet and Lifestyle in Women with Breast Cancer: An Update Review of Related Research in the Middle East

**DOI:** 10.1089/biores.2018.0004

**Published:** 2018-05-01

**Authors:** Zainab Taha, Sakina E. Eltom

**Affiliations:** ^1^ Department of Health Sciences, College of Natural and Health Sciences, Zayed University, Abu Dhabi, United Arab Emirates.; ^2^ Department of Biochemistry & Cancer Biology, Meharry Medical College, Nashville, Tennessee.; ^3^ Center for Women's Health Research, Meharry Medical College, Nashville, Tennessee.

**Keywords:** Arab women, breast cancer, chemoprevention, diet, Middle East, nutrition

## Abstract

Breast cancer is the most common malignancy among Arab women in Eastern Mediterranean Region (EMR). The incidence of breast cancer has substantially increased in recent years among this women population, especially those younger than 50, and the incidence is expected to double by 2030. Considerable experimental evidence supports the potential role of dietary habits and lifestyle in cancer etiology and cancer prevention. In this review we examined the literature for evidence to link dietary choices and the rise in incidence and mortality of breast cancer among women in EMR. A literature search was conducted in PubMed and Ovid MEDLINE databases up to December 2017. The search terms used are breast cancer prevalence, breast cancer incidence worldwide, breast cancer and: nutrition, protein intake, vitamin D intake, fat intake, phytoestrogens, EMR, Arab, Middle East, Gulf countries, the UAE Arab women, breast cancer risk, diet, and chemoprevention. We found evidence to suggest that there is an alarming epidemic of obesity among women in most of the EMR countries, especially Gulf Cooperation Council (GCC) countries. The rise in the new breast cancer cases among women could be attributed to excess body weight. Their dietary pattern, which correlates with obesity, can be an important factor in the etiology of cancer. Although very few studies were found to support a direct causal relationship between obesity and breast cancer in the EMR, circumstantial evidence clearly points to the possible role of the epidemic, obesity, in this population and the startling rise in cases of breast cancer. Well-designed and systematic studies are urgently needed to confirm these associations and to elucidate potential mechanisms. More urgently, calls to action are needed in many sectors and at all levels of society, to establish intensive strategies for reducing obesity and promoting an overall healthy diet. Continued and expanded research on diet, lifestyle, and breast cancer risk is urgently needed to build the foundation for future progress in evidence-based public health efforts.

## Introduction

It is well documented that the risk factors for breast cancer are mainly related to hormones through their influence on growth of the mammary glands. The findings from the collaborative analysis of data that were collected from a total of 47 epidemiological studies conducted in 30 countries have contributed tremendously to shed light on factors that are associated with breast cancer. These findings were published by the Collaborative Group on Hormonal Factors in Breast Cancer and they have established an important role for childbearing and breastfeeding on breast cancer risk.^1^ However, dietary factors can play a role in the etiology of breast cancer.

The Eastern Mediterranean region (EMR) encompasses 22 countries spanning from Morocco in the west to Pakistan in the east, and contains a population of almost 600 million people. Like many other developing regions, the burden of disease in the EMR has shifted in the past four decades from primarily communicable diseases to noncommunicable diseases, such as cancer and cardiovascular diseases. This shift has been attributed to the escalating obesity rate, which is becoming a major public health problem in the region and has been associated with the population shift toward a westernized diet.

In this review, we are examining the literature for evidence to link the changes in lifestyle and dietary choices (including obesity, low fruit and vegetable intake, low level of physical activity, etc.) and the rise in incidence and mortality of breast cancer among women in the Middles East. Conversely, we will examine the literature for dietary intake by women in this region of certain nutrients that will have chemopreventive roles against breast cancer.

## Methods

A literature search was conducted in PubMed and Ovid MEDLINE databases up to December 2017. The search terms used: breast cancer prevalence, breast cancer incidence worldwide, breast cancer and: nutrition, protein intake, vitamin D intake, Fat intake, phytoestrogens, EMR, Arab, Middle East, Gulf countries, the UAE Arab women, breast cancer risk, diet, chemoprevention. A list of 54 articles were deemed to meet the criteria and used for the review analysis. Additionally, we used GLOBOCAN worldwide estimates of cancer incidence and mortality produced by the International Agency for Research on Cancer (IARC).

## Results

### Breast cancer incidence and mortality in EMR

Most of cancer deaths in WHO EMR are attributed to lung and breast cancer.^2^ By and large, the breast cancer is the most common cancer among women in all countries of EMR,^3^ ranking first as the most frequent carcinoma diagnosed among women, (summarized in Table 1). Data from GLOBOCAN (2014) showed that the ratio of mortality:cancer incidence in EMR mounting to more than 42% compared with <19% in the United States and 31% worldwide (Table 2). In women, the average breast cancer incidence in this region is 33.9% of total women cancers, with world age-standardized rate of 41.9 per 100,000 and 24% mortality rate (Table 3).

**Table 1.** **The Three Most Frequent Cancers in Women by Total Number of Cases, in Eastern Mediterranean Region by Country, 2012**

Country	First	Second	Third
Qatar	Breast	Colorectum	Thyroid
United Arab Emirates	Breast	Thyroid	Cervix uteri
Bahrain	Breast	Colorectum	Cervix uteri
Kuwait	Breast	Colorectum	Thyroid
Saudi Arabia	Breast	Colorectum	Thyroid
Libya	Breast	Colorectum	Cervix uteri
Lebanon	Breast	Colorectum	NHL
Islamic Republic of Iran	Breast	Colorectum	Stomach
Oman	Breast	Thyroid	Colorectum
Tunisia	Breast	Colorectum	Cervix uteri
Jordan	Breast	Colorectum	Thyroid
State of Palestine	Breast	Colorectum	Thyroid
Egypt	Breast	Liver	NHL
Syrian Arab Republic	Breast	Colorectum	Leukemia
Morocco	Breast	Cervix uteri	Colorectum
Iraq	Breast	Leukemia	Brain, nervous system
Pakistan	Breast	Lip, oral cavity	Cervix uteri
Yemen	Breast	Leukemia	Thyroid
Djibouti	Breast	Cervix uteri	Ovary
Sudan	Breast	Cervix uteri	Ovary
Afghanistan	Breast	Cervix uteri	Corpus uteri
Somalia	Breast	Cervix uteri	Esophagus
Total EMR	Breast	Colorectum	Cervix uteri

GLOBOCAN 2012 data are used, which are based on estimates of cancer incidence and mortality. This table is extracted from table 1 of Kulhanova et al.^3^

EMR, Eastern Mediterranean Region; NHL, non-Hodgkin lymphoma.

**Table 2.** **Breast Cancer: Estimated Incidence, Mortality, and Prevalence in Eastern Mediterranean Region Compared with Other Regions and Worldwide in 2012**

Estimated numbers (thousands)	Cases	Deaths	5-Year prevalence
World	1671	522	6232
More developed regions	788	198	3201
Less developed regions	883	324	3032
WHO EMR	**99**	**42**	**348**
United States	233	44	971

Extracted from GLOBOCAN 2012, WHO, IARC. Bold emphasizes values in EMR relative to other regions in the world.

IARC, International Agency for Research on Cancer.

**Table 3.** **Population Fact Sheet: WHO Eastern Mediterranean Region: Estimated Incidence, Mortality, and a 5-Year Prevalence of Breast Cancer Compared with All Cancers in Women**

	Incidence			Mortality			5-Year prevalence		
	*N*	%	ASR^a^ (world)	*N*	%	ASR^a^ (world)	*N*	%	Proportions
Breast cancer	**99,284**	**33.9**	**41.9**	**42,228**	**24**	**18.6**	**347,565**	**47.4**	**171.8**
All cancers, excluding nonmelanoma skin	292,677	100	126.2	176,139	100	79.4	732,587	100	362.1

Incidence and mortality data for all ages. A 5-year prevalence for adult population only. Bold emphasizes breast cancer values compared to the values of all cancers.

^a^ ASR (world) and proportions per 100,000.

ASR, age-standardized rate.

An earlier report (2002) from the IARC showed breast cancer to be the most common cancer among females in all the six Gulf Cooperation Council (GCC) countries,^4^ with incidence rates ranging from 14.5% of all women cancers in Oman to almost one-third in Kuwait and Bahrain. Based on these data, the report classified Kuwait, Bahrain, and Qatar as high-incidence areas and the United Arab Emirates, Saudi Arabia, and Oman as low incidence areas of the GCC region.

A later analysis (2009) of cancer data from the GCC countries showed that breast cancer was still the most common cancer among women with the same trend of incidence rate among these countries, ranging from 16.1% in Oman to 35.4% in Bahrain. The age-standardized incidence rate (per 100,000 population) was the highest in Bahrain (46.4), followed by Kuwait (44.3), and Qatar (35.5), whereas United Arab Emirates (19.2), Oman (14.4), and Saudi Arabia (12.9).^5^ Peculiarly, most breast cancer cases reported were among women younger than 50 years of age.^6^

Studies from Saudi Arabia reported a steady increase in the prevalence of breast cancer diagnosis during 1990–2010.^7,8^ One study reported 1152 cases in 2008 increasing to 1308 cases in 2009 and 1473 in 2010, with the average age at the diagnosis of breast cancer at 48 years of age.^7^ Another study published in 2012, on breast cancer in Saudi Arabia confirmed that between years 2000 and 2007, there was a steady increase in the incidence of malignant breast carcinoma from 23.5% to 47.2%.^8^ In Oman, 1294 cases of breast cancer were reported in 2014 and 53.5% of them were below 50 years of age.^9^

In addition to being a country of high incidence rate, a study showed that the breast cancer in Kuwait appears to be biologically of a very aggressive type (overexpression of Her-2 and lack of estrogen receptor expression), accounting for the high mortality rate that occurs in ∼43% of breast cancer patients.^10^ This study also identified some of the risk factors of breast cancer among these women to include high body mass index (BMI), lack of regular exercise, early age at menarche, late age at first pregnancy, hormonal therapy, and frequent consumption of carbohydrate, sugar, animal fat, and hydrogenated vegetable oil (margarine) with low intake of fresh vegetables and olive oil.^10^

According to the Health Authority Abu Dhabi (HAAD) report in 2008,^11^ breast cancer is one of the leading cancers in the United Arab Emirates; considering cancer is the third top leading cause of death in the United Arab Emirates. Most cases of breast cancer in the UAE reported between 2005 and 2007 were among Arab women, and 64% of breast cancer patients in United Arab Emirates present with advanced cases of regional node involvement and/or metastases compared with only 20% in the United States.^11^

In a study comparing breast cancer picture in North African countries, including Morocco, Algeria, Tunisia, Libya, and Egypt to that in Western countries, the results showed that epidemiological features of breast cancer were different. North African countries have higher proportion of young patients. Notably, the incident rates of breast cancer among younger patients are much higher compared with the older generation. That difference was related to combined factors, including the rapid change in the lifestyle, diet, and emerging reproductive behaviors, such as declining in number of children, the increased age at first pregnancy, and shorter duration of breastfeeding.^12^ For instance, in Egypt, total fertility rates were 6.0 children per woman in 1988 compared with 3.1 in 2008 and in Morocco 5.7 in 1980 compared with 2.5 in 2003.^12^

### Diet and physical activity in EMR

Diet and physical activity are important risk factors and one-third of the breast cancer cases could be prevented by lifestyle intervention through reducing alcohol intake, increasing the fruit, vegetables, and vegetable oil intake in the diet, and increasing physical activity level.^13^ According to reports from the WHO and IARC, approximately one third of deaths from cancer are attributed to five leading lifestyle and dietary choices, including high BMI, low fruit and vegetable intake, low level of physical activity, tobacco, and alcohol consumption.^14,15^ Although the tobacco and alcohol consumption is not much of a problem among women in the Middle East region, because of religious and societal traditions, the remaining factors are profoundly contributing to the increased deterioration in the health of women population in this region, more specifically to the rising incidence of breast cancer and its dismal outcome.

With the sudden transition and the rapid change in the economy, Eastern Mediterranean (Middle Eastern) people change their traditional diet to a more westernized diet that has high energy and fat-rich food intake. They also adopted eating out habits, and an increase in food portion sizes in most countries in the EMR.^16^ It is very apparent that with the rapid improvement in the economy in these countries, people are becoming affluent and are consuming diets high in saturated fat, cholesterol, salt, and refined carbohydrates, and low in polyunsaturated fats and fiber, along with a marked sedentary lifestyle and increased stress.^17^ Low intake of fruit and vegetables was reported in children and adolescents, as well as in adults in many countries in the EMR. In general, the intake was lower than the recommended daily allowance (>400 g) in all age groups and in both genders.^16^

### Breast cancer and diet/physical activity in EMR

Breast cancer is the most frequently diagnosed cancer among women in all 22 countries of the EMR^3^ and obesity is a risk factor that is strongly associated with breast cancer. Alarmingly, overweight and obesity are at an extreme high level in many EMR countries, including Qatar, Kuwait, United Arab Emirates, Bahrain, and Saudi Arabia (Table 4). These countries are among the top 10 countries with the highest rates of obesity worldwide.^18^ An increase of 4980 (2%) of new cancer cases among adult men and 17,018 (7%) among adult women in the region were attributed to excess body weight.^19^ Dietary pattern, which correlates with obesity, can be an important cause of cancer, and lack of physical activity can also contribute to postmenopausal breast cancer. The increase in utilizing car transportation, the reduction in physically demanding occupations, the increasing use of domestic helpers, and increasing degree of sedentary lifestyles, all have contributed to the dramatic decline in physical activity in the EMR.^16^ The level of physical inactivity is one of the highest worldwide, reaching almost 70% in Saudi Arabia.^20^

**Table 4.** **Overweight-Obesity Prevalence (Female/Male) in Some Gulf Cooperation Council Countries**

BMI	Bahrain	United Arab Emirates	Qatar	Kuwait	Oman	Saudi Arabia
>25 (%)	67.4/61.0	69.7/66.9	64.1/57.9	79/69.5	47.8/43.4	63.8/63.1
>30 (%)	35.2/21.2	39.4/24.5	29.3/17.4	52.9/29.6	14.8/7.7	33.8/23.0

BMI >25 is overweight and >30 is obese. Source: Data from WHO (2006).^55^

BMI, body mass index.

### Dietary intake and breast cancer chemoprevention

In general, dietary intake of certain nutrients can play major roles in modifying breast cancer risk. Several studies investigated the relation between some nutrients and breast cancer.

### Dietary β-carotene and vitamin E and breast cancer

Diet rich in β-carotene and vitamin E, such as fruits, vegetables, and vegetable oil, is considered protective against breast cancer risk. Vitamin E is an antioxidant that was shown to decrease the number of carcinogen-induced mammary tumors in laboratory animals. Altogether, β-carotene and vitamin E are considered the strongest protective factors among numerous micronutrients.^13^

### Vitamin D and calcium intake and breast cancer

Several studies suggested that there was no association between vitamin D and calcium intake and breast cancer risk.^21,22^ However, a case–control study in Saudi women showed that there is an inverse association between serum 25(OH)D (the active metabolite of vitamin D) concentrations and breast cancer risk.^23^

### Dietary fat and protein intake and breast cancer

An association was found between protein intake and breast cancer risk, as the high intake of red meat, fresh and processed, increases the risk of breast cancer, whereas the high intake of soy food and skim milk reduces the risk.^24^ A prospective cohort study among women showed that high consumption of red meat during adolescence was associated with premenopausal breast cancer. Whereas, replacing red meat with other dietary protein sources in adolescence could decrease the potential risk of premenopausal breast cancer.^25^

On the other hand, some studies pointed out that there is a weak positive association between saturated fat intake and breast cancer risk, especially among postmenopausal women who never used hormone therapy.^26^ In general, there are rather inconsistent reports on the association between fat intake and breast cancer.^27,28^ For instance, outcomes from some case–control studies have suggested a positive association between saturated fat intake and risk of breast cancer, whereas other studies demonstrated a reduced risk from total and polyunsaturated fat intake.^29^ Results from the nurses study which compared nurses who consumed low-fat diets with nurses who consumed high-fat diets have concluded that no relationship existed between the amount of fat consumed and the risk of breast cancer.^30^ The Willett's review of epidemiological studies of the relationship between diet and breast and colon cancers suggested that there was a null or weak association between dietary fat and breast cancer.^31^ With regard to Arab women, a case–control study investigating the association between dietary fat and breast cancer in Saudi women, reported a significant positive association between the intake of fats, protein, and calories and risk of breast cancer.^32^

In comparison, dietary patterns and its association to breast cancer have been investigated in several studies in the west. Among all the dietary factors that were studied, alcohol consumption has been found frequently to be significantly associated with breast cancer.^33^ Plant-based diets, including fruits and vegetables were associated with a reduction in breast cancer risk as compared with food rich in fat and meat. This could be attributed to the role of fiber in fruit and vegetables that bind with estrogens and lead to excretion.

### Phytoestrogen compounds and breast cancer

Phytoestrogens, natural compounds that share chemical structure similar to estrogens are found mainly in plants such as soy beans, red and yellow fruits and vegetables, flaxseeds, and whole grains.^34^ Several classes of phytoestrogens exist, which include isoflavones, stilbenes, coumestans, and lignans.^35^ Resveratrol, daidzein, quercetin, and genistein represent four of the most commonly ingested and most widely studied phytoestrogens.^36^ The average daily consumption of phytoestrogens among East and Southeast Asia is estimated to be around 20–50 mg/day; whereas the daily consumption in the United States and Europe, respectively, is only 0.15–3 and 0.49–1 mg/day.^37^ According to epidemiological studies, plasma levels of isoflavone in Japanese men is ∼2 μM in contrast to ∼5 nM in Finnish men; however, local tissue phytoestrogen concentrations are suggested to be two to three times higher than plasma levels.^36^ Epidemiological studies provided evidence that phytoestrogens have an effect in reducing the incidence of breast cancer, especially among Asians who have high dietary intake of isoflavonoids through their daily diet.^38–40^

The notion that dietary and lifestyle factors may be partially responsible for the low breast cancer risks detected in Asian women is supported by observations in Asian women who immigrate to Western countries. The second- and third-generation descendants of women who migrated from Asia to Western countries have breast cancer risks similar to those of women in the host country, suggesting that lifestyle and not genetic factors explains the low breast cancer risk of women in Asia.^36,41^ However, a review of 21 case–control and 15 prospective studies concluded that there is no clear evidence that phytoestrogen intake influences the risk of developing breast cancer.^36,42^

### Mediterranean diet and chemopreventive role on breast cancer

The dietary consumption of fruit, vegetables, grains, legumes, olive oil, and the moderate intake of red wine provide high levels of different polyphenols and plant bioactive compounds that can serve as antioxidants, anti-inflammatory, and antitumor.^43^ Mounting evidence points to the beneficial and preventive role of the Mediterranean diet (MD) in the onset of cancer and other diseases associated with increased level of inflammation, oxidative damage, and angiogenesis. A recent meta-analysis of all the observational studies regarding the adherence to the MD in relation to cancer risk^44^ showed that the MD is associated with a significant reduction of overall risk of cancer incidence and mortality by 10%. A meta-analysis by Schwingshackl and Hoffmann did not confirm an effect of the MD on the risk of breast cancer; however, a subgroup of case–control study showed that the risk of breast cancer could be reduced by 18% in women adhering to the MD. Chemoprotective effect of the MD against breast cancer seemed to depend on individual's characteristics and potential risk factors, such as obesity, physical activity, smoking, age at menarche, and menopausal status. Notably, the beneficial effect of the MD on breast cancer risk is observed only in normal weight, nonsmoking women and in women who did not present an early menarche (<12 years of age).^45^

### Nutrition and breast cancer management

Diet and nutritional status are not only important factors in the etiology of breast cancer, but they are—major determinants of prognosis and treatment outcome in breast cancer patients. Furthermore, scientific observations support the idea that dietary supplement can prevent breast cancer recurrences.

Not limited only to breast cancer, malnutrition is frequently manifested in cancer patients, even at the time of diagnosis. Its incidence varies between 31% and 87%, depending on the stage, type, treatment, and the individual patient.^46,47^ Weight loss can occur, as a result of elevated energy requirements, low energy intake, or impaired absorption of nutrients. In cancer patients, undernutrition may be due to various factors. Inflammation and catabolism created by the tumor can result in muscle wasting and weight loss,^48^ whereas tumor gastrointestinal obstruction can impair food intake and absorption, as a result of dysphagia, pain, and vomiting. In addition to this, the side effects of anticancer treatment, such as anorexia, nausea, vomiting, oral and intestinal mucositis with dysphagia, diarrhea, hemorrhoids, anal fissures, and modifications in smell and taste affect not only the total energy intake, but also the nutrient absorption, compromising nutritional status. Furthermore, the poor psychological state of cancer patients can affect their energy intake.^49,50^ Malnutrition can impact disease progression and survival in cancer patients. Substantial studies have shown that weight loss in cancer is associated with poor prognosis, poor quality of life, lower activity level, increased treatment-related adverse symptoms, and reduced tumor response to therapy.^51^ Weight loss at diagnosis has also been associated with shorter failure free and overall survival (OS), being identified as an independent prognostic factor. However, when patients stop losing weight they have better OS.^52^

## Discussion

Socioeconomic development is usually associated with increasing wealth, changing lifestyle, and disease pattern leading to increase in life expectancy. This association of changes, known as epidemiological transition, is very well demonstrated in the Middle East, especially in the Gulf States, which have experienced rapid socioeconomic changes during the last four decades. As food habits trail changes in lifestyle, there has been a dramatic shift from the indigenous traditional healthy diet, which was based on whole wheat flour, fish, milk, and dates toward an affluent diet, which is rich in total calories, meat, saturated fat. and refined carbohydrates. This change in dietary habits, taking place in parallel with reduced physical activity, may have contributed to the increase in the number of breast cancer cases in the regions.^53^ Dietary fat has been proposed as one of the etiologic factors for breast cancer.^28^ However, the relationship between fat intake and the risk of breast cancer has been examined in a number of case–control and cohort studies. The findings reported in the literature are not conclusive enough to establish a pattern for the real cause of the disease.^30,31^

## Conclusions and Implications

Globally there is sufficient evidence to strongly support the notion that overweight, obesity, and reduced physical activities have direct causal relationship to breast cancer. Although very few studies are available to directly document such a relationship in the Arab world, circumstantial evidence clearly points to the possible role of the epidemic obesity in this population and the startling rise in cases of breast cancer. Well-designed and systematic studies are urgently needed to establish these associations. Although this review has shown that dietary habits and lifestyle have an influence on the incidence of breast cancer, it is still evident that the main etiological factors are hormone related. McDonald et al. have documented that the incidence rates of breast cancer in women who immigrated to Canada has remained the same as it is in their control groups back home despite acquiring a Western lifestyle.^54^ Calls to action are urgently needed in many sectors and at all levels of society, to establish intensive strategies and polices for reducing obesity and promoting physical activity. Strong and persistent efforts are also needed to promote an overall healthy lifestyle with special emphasis on diet that is rich in fruit and vegetables and whole grains and low in red meat and saturated fats. Continued and expanded research on diet, lifestyle and breast cancer risk is urgently needed to build the foundation for future progress in evidence-based public health efforts in this region of the world.

## Abbreviations Used

BMI: body mass index
EMR: Eastern Mediterranean Region
GCC: Gulf Cooperation Council
IARC: International Agency for Research on Cancer
MD: Mediterranean diet
OS: overall survival
